# Application of Nanomaterials in Restorative Dentistry

**DOI:** 10.7759/cureus.33779

**Published:** 2023-01-14

**Authors:** Rutvik Mandhalkar, Priyanka Paul, Amit Reche

**Affiliations:** 1 Dentistry, Sharad Pawar Dental College and Hospital, Datta Meghe Institute of Medical Sciences, Deemed to be University, Wardha, IND; 2 Public Health Dentistry, Sharad Pawar Dental College and Hospital, Datta Meghe Institute of Medical Sciences, Deemed to be University, Wardha, IND

**Keywords:** nanoparticles, caries, anti-bacterial, composite resin, restoration

## Abstract

Dental composite resins are widely popular restoratives, as, when using these tools to restore the tooth, only the infected and affected carious structures are removed. This allows the patient to retain a greater quantity of their natural tooth structure than they would have using conventional principles of cavity preparation. Nanomaterials are a new concept concerning the manipulation of materials on an atomic or molecular level. However, on a nanoscale, the chemical, biological, and physical properties of an atom vary compared to the properties of its naturally occurring compound form. The main idea of shifting focus to the inclusion of nanomaterials is to aid in the detection, treatment, and prevention of the recurrence of a pathology (secondary caries). The primary aim of using nanomaterials in composites is to augment their strength, wear resistance, and microhardness. This usage also reduces polymerization shrinkage. Nanomaterials are capable of enhancing mechanical properties, life, and bond strength between dentin and restoration. This review aims to highlight different research studies and experiments that have been conducted on the use of nanomaterials in restorative dentistry in order to understand the versatility of these materials and their viability in practice.

## Introduction and background

Tooth restoration is one of the main procedures in dental treatment. It is concerned with the re-establishment of the normal structure of the teeth such that the treated or restored teeth mimic the nature and function of the original non-affected or non-infected teeth. The classic cavity preparation principles were established by the late Dr. G.V Black. His principles of tooth preparation aimed to guide an operator to prepare a cavity such that when the restoration is placed, it assumes the function of the tooth perfectly and stays inside the cavity for a long time and mimics the natural behavior and function of the tooth. The cavity prepared on the principles of Dr. Black was, for the most part of the endodontic history, filled using amalgam. Amalgam is an alloy of silver and mercury in a 3:7 ratio [1]. It was continuously improved by adding filler particles. Amalgam has been the principal restorative material used for most of endodontic history, but with the evolution of technology and knowledge, it has now been widely substituted with dental composite resins, a better, more versatile, aesthetic, and comparatively stronger alternative.

Dental composite resins are widely popular because they allowed operators to abandon the conventional principles of tooth preparation of Dr. Black for a far more conservative approach wherein only the infected and affected carious structures are removed; thus, in many cases, with the use of these resins, patients could retain a greater quantity of their natural tooth structures as compared to the use of conventional principles.

Dental composite resins are commonly composed of dimethacrylate monomers (Bis-GMA, TEGMA, and UDMA), along with multi-functional filler materials, which help enhance elastic modulus, increase strength and wear resistance, and decrease the polymerization shrinkage of the restoration [1]. These filler particles can be classified as macro fillers, micro fillers, micro-hybrid fillers, and nanofillers. Much like amalgam was improved using copper, composites were improved using various filler materials, one of which is nanofillers. This article aims to review the improvements made by modern nano-materials to dental composite resins compared to other filler materials.

Why nano-materials?

Nano-materials are used to manipulate materials on an atomic or molecular level. However, on a nanoscale, the chemical, biological, and physical properties of an atom vary compared to its naturally occurring compound forms [1]. Human tooth structures include enamel that is made of 96% hydroxyapatite of size ranging from 10nm to 200 nm [2]. Intertubular dentin is 2-5nm in thickness and 60nm in length. Enamel and dentin are connected by collagen fibrils that stretch over 20-75 nm in length [3]. These characteristics provide a solid ground for the basic need for research applications of nano-materials in dentistry. The use of nano-materials in dentistry is known as nano dentistry [4,5]. The main idea of shifting focus to the application of nano-materials is to aid in the detection, treatment, and prevention of the recurrence of pathology (secondary caries). The dawn of the digital age and increased patient needs and demands for aesthetics and functionality have posed a challenge to the field of engineered nano-materials [6]. Nanocomposites are biomaterials in which at least one phase shows dimensions in the nanometer range. The size of nano-materials ranges from 5nm to 100nm. The incorporation of such filler particles leads to color alteration and increases the flexural strength of the composite resin restoration [7]. Currently, a plethora of nano-materials is being experimentally studied to determine their ability to enhance products of wide-ranging disciplines. In dentistry, particularly restorative dentistry, nano-materials are used to manufacture nanocomposites [8], glass ionomer cement, and endodontic sealer. The main purpose of using nano-materials in composites is to augment their strength, wear-resistance, and microhardness, as well as to reduce the polymerization shrinkage [9]; however, polymerization shrinkage varies depending on the chemical structure and manufacturing process of the composite [10]. However, research shows that the optimum wt% of nanofillers should not be exceeded, as no improvement in mechanical properties can be achieved further [6]. Nano-materials are capable of enhancing the mechanical properties, life, and bond strength between dentin and restoration [11].

**Figure 1:**
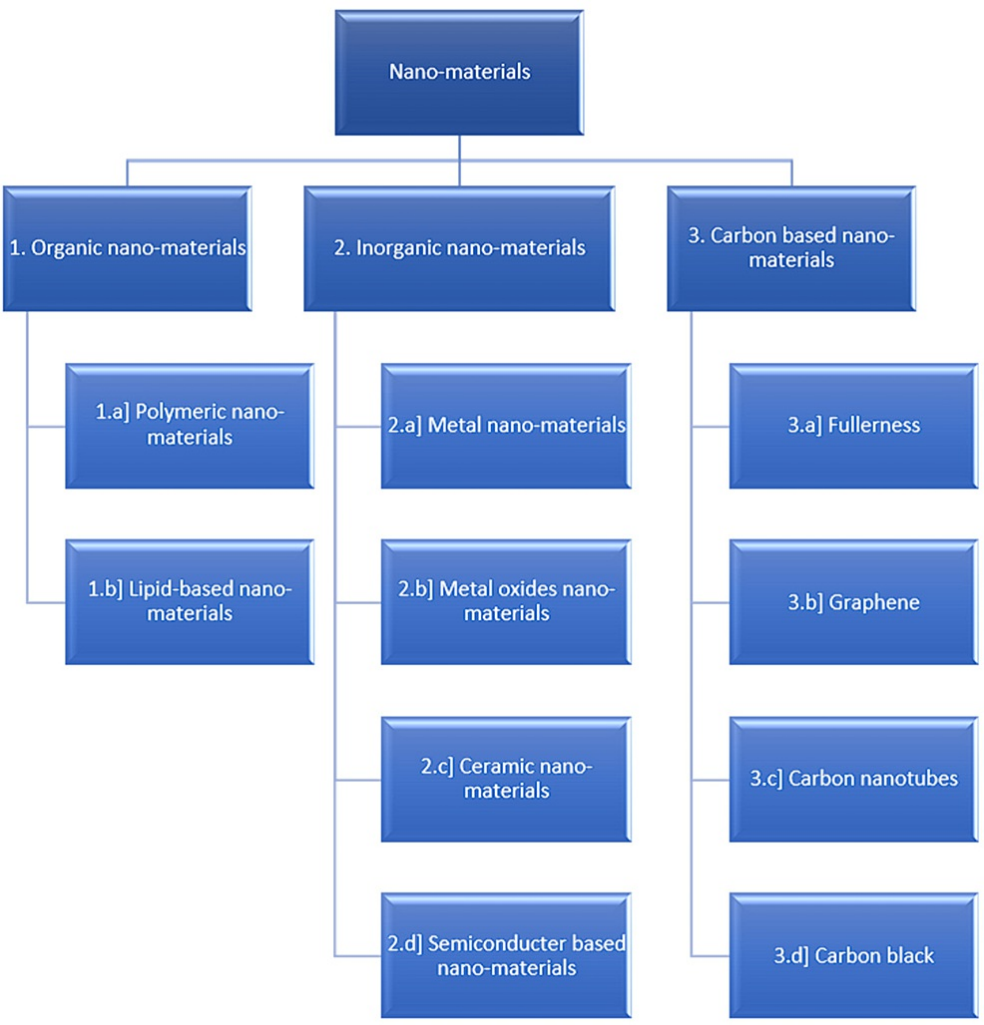
Classification of nano-materials.

The synthesis of nano-materials is mainly done in two ways: in the top-down method, a bulk of the material is reduced to nanometric measurements using various methods, and in the bottoms-up method, individual atoms are clumped together [11]. The classification of nano-materials is illustrated in Figure 1 [11]

## Review

Research methodology

**Figure 2:**
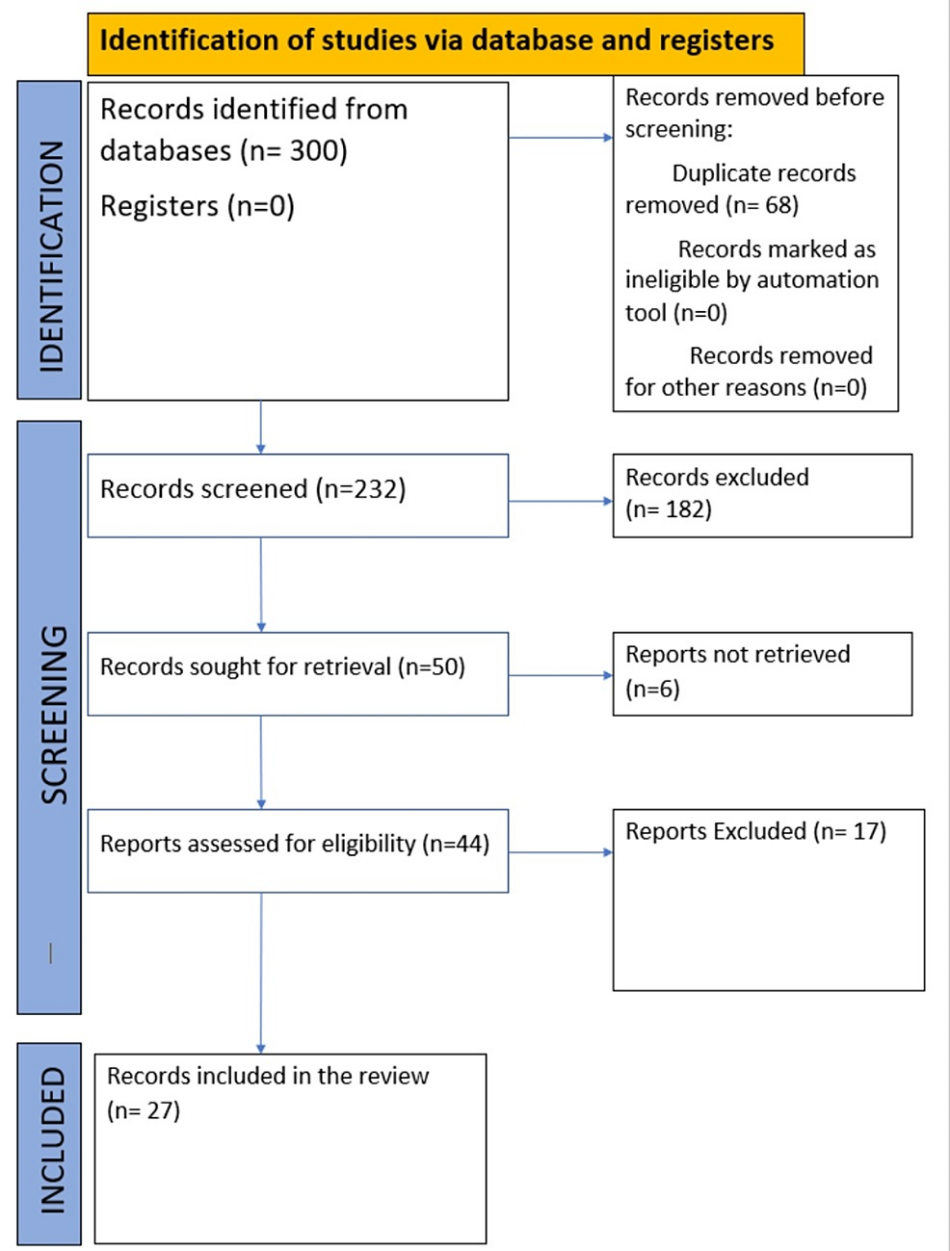
PRISMA flow diagram. PRISMA: Preferred Reporting Items for Systemic Reviews and Meta-Analyses

Primarily, articles and case studies were searched and obtained from the databases PubMed and Google Scholar. The inclusion criterion was studies that discussed the use of nanoparticles or the scope of nanoparticles in restorative dentistry only; further, the selected studies should also have discussed their results. The PRISMA flow diagram of the included studies is shown in Figure 2.

Discussion

The interaction of various nano-materials with resin restorations has exhibited the ability to improve various aspects of such restorations.

Xiao et al. [12] developed a bioactive multifunctional composite (BMC) using nanoparticles of NACP, 2-methacryloyloxyethyl phosphorylcholine (MPC), dimethylaminohexadecyl methacrylate (DMAHDM), and silver (AgNP) and investigated the effect of poly(amido amine) (PAMAM), which was added to the BMC. The focus of the study was on root caries and demonstrated excellent root dentin remineralization. This study successfully developed a bioactive multi-functional composite for Class V restoration. Along with remineralization, the developed BMC was protein-repellent and had anti-bacterial capabilities [12]. It concluded that BMC + PAMAM protects tooth root structures and is promising for Class I, Class II, and other types of tooth cavity restorations [12].

Ghahremani et al. [13] evaluated a color-modified heat cure resin that was enhanced with titanium oxide (TiO_2_) nanoparticles for tensile and impact strength. In this in vitro study, the researchers added TiO_2_ nanoparticles to a triplex hot heat-cure resin and blended them together. Subsequently, it was found that the reinforced group's strength is much greater, specifically 7 MPa more than the strength of the control group; this difference was statistically significant (p = 0.001). Thus, the study concluded that the addition of TiO_2_ by 1% wt to color-modified acrylic resin increased tensile and impact strength; however, this study did not consider any side effects this approach may have caused on the restorative resin and its restorative ability [13].

In a comparative study performed by Meena et al. [14], the researchers added marble powder to composite resin and compared its performance to composite added with nano alumina. They performed dynamic mechanical analysis and thermos-gravimetric analysis to compare the role of both components to the composite resin and its performance as a restoration. It was observed that marble dust-filled composites absorbed less water and had a lesser diffusion coefficient. Thermal stability of the nano-alumina-filled composite was also found to be lacking by 20% compared to composite filled with marble dust. The results drawn out from their analysis indicated that marble dust is a superior filler material as it showed better physical and mechanical properties compared to nano alumina; moreover, marble dust is a more economic and cheaper alternative to nano alumina [14]. However, further study is needed to see if restorations with marble dust-filled composites could have a long-standing effect on tooth biology.

Wang et al. [15] incorporated wrinkled mesoporous silica (WMS) with unimodal and bimodal fillers. The resin matrix this was mixed with was BisGmA/TEGDMA based. Bimodal WMS fillers comprising either WMS-Si90 or WMS-Si190 were used to create resin composites, with all of them permitting 60 wt% filler loading and surpassing the loading limits of unimodal WMS (35 wt%). This study found that bimodal filler-mixed WMS produced better mechanical properties compared to its unimodal filler counterpart [15].

In the study by Al-Mosawi [16], the researcher used ZnO nanoparticles, incorporated them into a composite resin, and evaluated their potential as an anti-bacterial agent for the oral cavity. He made his observations on agar at different concentrations such as 5%, 7%, and 10%; among all isolated bacterial species that demonstrated activity, the most vulnerable bacterial isolates were *S. mutans* and pseudomonas, respectively. These observations indicated that ZnO/NPs can be effective in secondary caries prevention [16].

Ai et al. [17] sought to discover a functional one-dimensional nanofibrous filler for composite resins with which effective reinforcing and excellent antibacterial activity can be achieved. By using calcium oleate as a precursor through the hydrothermal technique, hydroxyapatite (HA) nanowires were synthesized. These nanowires were coated in polydopamine (PDA) by soaking them in an aqueous dopamine solution. These were then treated with Ag nanoparticles. These AgNP-doped HA nanowires were mixed with Bis-GMA [50/50 w/w] and prepared through thermo-curing. These modified HA-PDA-Ag wires demonstrated great adherence with the Bis-GMA resin matrix and effectively reinforced the resin matrix. The resultant composite displayed high bactericidal activity without any display of cytotoxicity and served as an ideal nanofiller [17].

Paiva et al. [18] aimed to develop polyacid formulations through photo-reducing Ag nanoparticles in a polyacrylate solution of standard glass ionomer cement (GIC) in a single step, retaining antibacterial activity and evaluating the handling and mechanical qualities of the Ag-added GIC as compared to a conventional GIC. The modified restoration was tested against *S. mutans*. This study found that the AgNP-added GIC had excellent antibacterial activity. The restoration acted on the principle of diffusion, indicating Ag ion dissolution (oxidative) from the cement matrix; this led to the conclusion that silver nanoparticles-added GIC can stop caries and prevent biofilm growth on their surface [18].

Stewart [19] aimed to make the restoration itself antibacterial, which would make it capable of defending itself against the possibility of secondary caries. He incorporated octenidine dihydrochloride (OCT) in drug-eluting mesoporous silica nanoparticles (DMSNs). These were fabricated using the bottoms-up synthesizing method. OCT is stated to be highly biocompatible [19], and no microbial resistance has been discovered yet [20]. Using silica nanoparticles, the researchers developed a dental resin adhesive that was localized, that is, confined to the restored surface of the tooth, but long-time drug-releasing, thereby minimizing the systemic exposure. Their analysis, thus, proved that long-term prevention of secondary caries can be achieved through the restoration itself [21].

Yue et al. [22] developed a self-healing adhesive with anti-microbial and re-mineralizing abilities and evaluated the effect of involving microcapsules such as DMAHDM and the nanoparticles of NACP for the first time. Self-healing microcapsules were synthesized with polyurea-formaldehyde (PUF) shells that are composed of 10% DMAHDM and 20% NACP. The single-edge V-notched beam method was used to calculate fracture toughness, K_ic_, and crack-healing efficiency. Yue et al. [22] achieved three results with the newly developed adhesive resin: autonomous crack-healing, anti-microbial capabilities, and remineralization function through the use of calcium phosphate nanoparticles. The fabricated adhesive also reduced the colony-forming units of microcosm biofilms by a magnitude of four grades compared to the conventional control. The unique strategy of employing triple agents (self-healing microcapsules + DMAHDM + NACP) to mend cracks and suppress caries is predicted to be suitable for a variety of dental adhesives, cement, sealants, and composites [22].

Cao et al. [23] developed a nano Ag-filled resin. By using AgBr/BHPVP nanocomposites, any adverse effect on flexural strength and modulus was averted. Meanwhile, the Vicker’s hardness of the resin discs was greatly increased. There was a sustained Ag+ ion release without any impact from the anaerobic environment. The developed resin was suitable to be used to combat anaerobic cariogenic bacteria. It has a particularly potent antibacterial effect on *S. mutans*, which is achieved through the continuous release of Ag+ ions. The study concluded that the optimal concentration of AgBr/BHPVP should be 1.0 wt% in Bis-GMA/TEGDMA [23].

The antibacterial effect of ZnO nanoparticles is higher than that of AgNPs with respect to harmful actions against *S. mutans *[24-26]. Additionally, nanodiamonds also show promising results. It was found that 1wt% quaternized copolymer functionalized nanodiamond-reinforced resin composites were capable of inhibiting biofilm formation without causing any harm to the tooth structure [27]. Not only composites, nano-materials also have shown an excellent integration with GIC. GIC is inherently capable of releasing fluoride ions, which combat the secondary recurrence of caries [18]. Copper-doped GIC shows superior antibacterial properties and a reduced rate of collagen degradation. Renné et al. [28] showed that the addition of nanohydroxyapatite powder to GIC enhanced its fluoride ion-releasing capability. Another study concluded that hydroxyapatite nanoparticles are suitable for improving the mechanical and bactericidal properties of GIC [29].

Xie et al. [30] aimed to develop an adhesive that has three advantageous properties: calcium phosphate ion recharging, protein repellent, and anti-bacterial function. MPC and DMAHDM were combined to create an NACP-rechargeable adhesive with protein-repellent and anti-bacterial properties to combat biofilms and caries. The developed bioactive adhesive had strong protein-repellent properties and significantly reduced bacterial adhesion. With these three advantages, the adhesive could protect tooth structures as well as reduce biofilm and caries progression [30].

All these studies show that the addition of nano-materials to composite resins for restoration is advantageous to restorative dentistry (Table 1).

**Table 1:** Literature reviewed in the study. BMC: bioactive multifunctional dental composite NP: nanoparticle NACP: nanoparticles of amorphous calcium phosphate DMAHDM: dimethylaminohexadecyl methacrylate MPC: 2-methacryloyloxyethyl phosphorylcholine NP: nanoparticle TiO_2_: titanium dioxide ZnO: zinc oxide HA: hydroxyapatite AgNP: silver nanoparticles GIC: glass ionomer cement Ag+: silver ion OCT: octenidine dihydrochloride DSPs: dexamethasone sodium phosphate NACP: amorphous calcium phosphate AgBr: silver bromide BHPVP: cationic polymer nanocomposite

Author	Aim	Observations
Xiao et al. [12]	To study BMC through the nanoparticles of NACP, DMAHDM, MPC, and AgNP	The root dentin exhibited a remineralization effect.
Ghahremani et al. [13]	To add TiO_2_ nanoparticles to improve mechanical properties	Color-modified acrylic resin showed a higher tensile and impact strength.
Meena et al. [14]	To incorporate marble powder into a dental composite resin and compare its performance with that of nano alumina-reinforced dental composites	The addition of nano alumina (5% wt) improved compressive strength by 23.25% and hardness by 88.4%. Marble powder improved (5% wt) hardness by 51.27% and compressive strength by 21.2%.
Wang et al. [15]	To examine the effect of resin matrix reinforced with wrinkled mesoporous silica	The reinforced resin matrix has superior mechanical properties.
Al-Eisa et al. [16]	To study ZnO nanoparticles’ antibacterial properties as an inorganic antibacterial agent	Bacterial growth decreased by 85%.
Ai et al. [17]	To create a useful, one-dimensional nanofiber filler for composite resin restoration that may provide effective reinforcement as well as have a strong antibacterial property	NPs-loaded HA nanowires can be employed as effective reinforcements for composite resins since they have enhanced antibacterial properties.
Paiva et al. [18]	To develop polyacid formulations for AgNP-reinforced GIC and compare it to commercially available conventional GIC, as well as assess the handling and mechanical qualities of experimental ionomers	Increased composition of silver allowed for a feasible net setting of time and a 32% increase in compressive strength; the release of Ag^+^ ions induced E. coli inhibition zones.
Stewart et al. [19]	To evaluate the ability of an antimicrobial drug-releasing resin adhesive comprising octenidine dihydrochloride (OCT)-silica co-assembled particles (DSPs) in improving the biostability of composite restorations bonded to dentin and maintain their interfacial fracture toughness	The resin adhesive expanded restoration life and can be used with other medical tissue interfaces for releasing drugs. The adhesive inhibits *S. mutans* but increases saliva degradative esterase activity.
Yue et al. [20]	To develop the first antibacterial and re-mineralizing self-healing adhesive, evaluate the impact of integrating microcapsules, DMAHDM, and NaCP NPs	With this adhesive, biofilm and acid production were reduced to 1/100th of that in standard resin. The product achieved crack healing and K_ic _recovery of 67%.
Cao et al. [23]	To incorporate AgNPs into composite resin	AgBr/BHPVP demonstrated dual bactericidal capabilities and a long-standing antimicrobial effect.

## Conclusions

The developments of different products using nanoparticles in the articles reviewed in this study proved that the demand and functions of nano-materials in restorative dentistry and dental practices are vast as a whole. The addition of various nanoparticles not only augmented the strength and life of restorations but also resisted secondary caries incidence by attributing antibacterial properties to the restorations. Besides, resin adhesive showed potential beyond assistance in restoration, functioning as a long-term local drug delivery tissue interface. Other than silver and titanium, nanoparticles have shown excellent biocompatibility with tooth; however, the level of integration with composite resin varied, although favorable results were obtained. While the addition of nanoparticles is certainly advantageous, the process of commercially manufacturing restorations infused with nanoparticles scale has not been thoroughly researched. An in-depth investigation needs to be conducted on the potential of the commercial manufacturing of nanoparticle-infused restorations, their competitive pricing on the market, and their availability to the general public while ensuring that the price of restoration as a medical service is not significantly impacted. Nanoparticles showed promise in various aspects of life; as such, as a new technology, nanoparticles have been researched extensively for their application in everyday life. This article reviewed the published studies on the use of nanoparticles in restorative dentistry to highlight the significance of this up-and-coming technology to the wider audience. While more clinical trials are needed to truly make this technology commercial, nano-materials still show the potential to emerge as a new-generation restorative material in the field of dentistry.
